# A Doença de Chagas: Seria o Barbeiro o Único Culpado?

**DOI:** 10.36660/abc.20210290

**Published:** 2021-07-14

**Authors:** Abilio Augusto Fragata

**Affiliations:** Instituto Dante Pazzanese de Cardiologia Laboratório de Doença de Chagas São PauloSP Brasil Instituto Dante Pazzanese de Cardiologia - Laboratório de Doença de Chagas, São Paulo, SP – Brasil

**Keywords:** Doença de Chagas/história, Trypanosoma Cruzi, Cardiomiopatia Chagásica, Miocardite, Mortalidade

## Preâmbulo

Descrita por Carlos Chagas em 1909, a enfermidade é causada por um parasita unicelular (*Trypanosoma cruzi*). Ao longo da história, o mecanismo principal de transmissão se deu através do contato das fezes de um inseto conhecido popularmente no Brasil como “barbeiro”, “chupão” e “chupança”, entre outras denominações, que estejam contaminadas com este parasita. Os “barbeiros” não nascem com o parasita em seu intestino, mas o adquirem quando se alimentam com o sangue de algum mamífero infectado (a doença só acomete mamíferos). Ao se alimentarem, estes insetos eliminam fezes, que contém os parasitas que podem contaminar a ferida da picada, propiciando a sua entrada no organismo, dando início a doença. Os “barbeiros” fazem seus ninhos principalmente nas rachaduras das paredes de barro das casas de “pau a pique”, muito comuns em várias regiões do Brasil, principalmente nos sertões do Nordeste. Outros mecanismos de transmissão podem ocorrer: transfusão de sangue, doação de órgãos, da mãe para o filho, ingestão de alimentos contaminados etc.

Na fase aguda da doença, as queixas podem ser leves ou mesmo não existirem. Podem surgir manifestações gerais, como febre baixa e mal-estar, ou mais graves, como falta de ar e inchaço.^1^ Como a picada do barbeiro é mais frequente na face, pode ocorrer a tumefação das pálpebras, conhecida como sinal de Romaña. Com o passar dos anos, geralmente uma década ou mais, em um terço dos pacientes aparecem queixas cardíacas, como cansaço, falta de ar e inchaço no corpo (insuficiência cardíaca).^2^ Em alguns casos, o sistema elétrico do coração pode apresentar comprometimento, com o aparecimento de alterações do ritmo cardíaco, desmaios e até morte súbita. Também podem ocorrer dilatações do esôfago e do cólon, com progressiva dificuldade para engolir alimentos e defecar.^1^

Desde os seus primeiros exemplares, que datam de 1948, os Arquivos Brasileiros de Cardiologia têm registrado o avanço no conhecimento médico da especialidade e a constatação de como os diversos pesquisadores da época, mesmo com poucos recursos técnicos, expunham um raciocínio clínico brilhante. Os primeiros dez artigos sobre doença de Chagas, nesta revista, datam de 1948 a 1958.^3^^-^^12^

**Figura 1:**
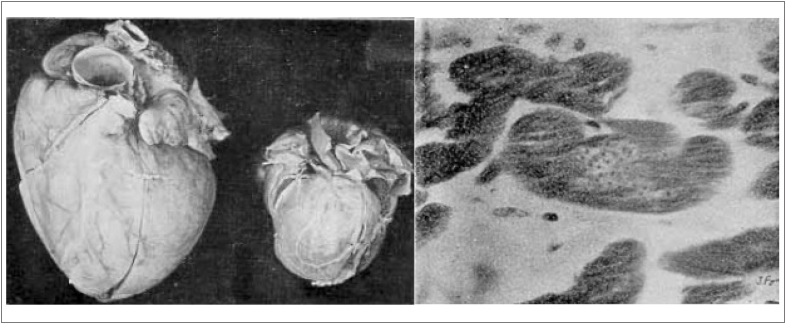
(A) À esquerda, coração do caso 26; à direita, coração normal. Na radioscopia: grande coração miogênico, parado quase totalmente. (B) Aglomerado parasitário no interior da fibra cardíaca (S. cruzi).^3^^,^^5^

Ao final da década de 1940, as figuras incluídas nos artigos já refletiam o interesse didático e pioneiro dos Arquivos Brasileiros de Cardiologia, como estas publicadas em 1948 e 1949 (Figura 1).

## Esta é minha história

Minha mãe deixou a mim e meus irmãos, ainda como ovos, em um lugar escuro com cheiro de barro, à nossa própria sorte. Quando saí do ovo, percebi que muitos dos meus irmãos também já haviam saído, mas outros não esboçavam qualquer reação. Mais tarde entendi que eles jamais saíram. Não sei quem é minha mãe, nem meu pai.

Ainda pequenino, explorando o território onde nasci, vi que meu ninho encontrava-se em uma pequena rachadura da parede de uma casa muito estranha. Ela era feita com taquaras entrelaçadas e recheadas com barro vermelho. Sentia-se um cheiro esquisito, mistura de suor e lenha queimada. Havia vários objetos bem envelhecidos: uma lamparina apagada sobre uma prateleira de madeira, quase podre, perto da porta escancarada, que era feita de pedaços de tábuas, com várias fendas. Havia um fogão com lenha em brasa, em cima do qual estava uma panela com pouca coisa, nada além de água fervendo e alguns pedaços de mandioca. Encostada na parede, bem embaixo do meu ninho, podia ver uma cama de casal com remendos nos quatro pés e na cabeceira, ameaçando ruir. Em cima dela, um colchão esfarrapado, coberto com um lençol também roto no qual se via tufos de palha saindo pelos rasgos. Na outra parede, uma janela aberta e embaixo dela uma mesa, igualmente em ruínas, cujas quatro pernas ameaçavam despedaçar. Não havia toalha, mas somente duas latas de ervilha vazias que serviam de copo e xícara. Ao lado, dois pratos totalmente lascados e uma colher bem gasta em cima de cada um deles. O chão era inteiramente de terra, no qual era possível ver pedaços de madeira e restos de comida, além de respingos do fogão de lenha. O teto era feito de palha seca com muitos buracos, que permitiam a entrada de raios de sol. Não havia ninguém na casa. Olhando para este cenário, achei meu ninho até bastante confortável.

Muitos dos meus irmãos já haviam saído e não soube mais deles. Aqueles que, como eu, ali ficaram, achávamos o ambiente repleto de tristeza. Não tínhamos sequer ideia de quem éramos. Fiquei sabendo, tempos depois, que éramos insetos conhecidos pelo nome de “barbeiro”.

Deixando meu ninho, comecei a explorar a casa e saí pela janela, temeroso do que poderia encontrar. O sol era escaldante e não havia uma brisa sequer para refrescar. O chão era de terra vermelha, e raramente se via um pouco de mato com folhas amareladas e distorcidas pelo calor. Pensei que os habitantes não sentissem sede, pois não havia vestígios de água. Pouco depois, ouvi um ruído e vi um cachorro muito emagrecido, língua de fora, andando indolente por aquele chão tórrido. Percebi que ele mal tinha forças para andar e latir ao mesmo tempo. Ele foi se aproximando e eu apavorado me escondi em uma rachadura que encontrei na parede do lado de fora daquele casebre. Fiquei imóvel ali enquanto o cachorro cheirava insistentemente, como se farejasse algo para comer. Em seguida, ele se afastou, entrou na casa e deitou no chão perto da porta, dormindo pesadamente, talvez para disfarçar a fome que sentia. Continuando minha exploração, avistei outro animal, muito estranho. Mais tarde, soube que o chamavam de gambá. Quando me viu, correu imediatamente em minha direção com a fisionomia horripilante, demonstrando sua intenção de, às minhas custas, saciar sua fome. Mais uma vez me vali do refúgio estratégico na fenda da parede, que desta vez se abria também para o lado de dentro da casa. Como já caía a noite, resolvi me recolher ao ninho e ficar lá em segurança. Confesso que adormeci, não sei por quanto tempo. Acordei assustado com o som alto que vinha de dentro da casa. Estava muito escuro e eu não conseguia enxergar. A seguir, uma luz muito fraca se fez presente e vi a lamparina acesa. Qual não foi meu espanto quando vi um homem, uma mulher e quatro crianças falando alto na casa. O homem era de estatura baixa, muito magro, tinha a pele do rosto enrugada e sofrida pela constante exposição ao sol e trazia nos pés um chinelo surrado. Vestia uma calça que mal chegava aos tornozelos, cheia de remendos e outros tantos rasgos que não suportavam mais costuras. Suas mãos, calejadas pela labuta diária na roça, eram incapazes de movimentos mais finos, manuseavam tudo com brutalidade. Trazia na boca um cigarro feito de palha, que espalhava uma fumaça malcheirosa, mas que lhe parecia confortante.

A mulher tinha um semblante resignado e igualmente emagrecida, pés descalços e calejados. Trazia na cabeça um pano que um dia deve ter sido branco, mas que agora era amarelado. Ele escondia quase todo o cabelo escuro e as mechas à mostra tinham a aparência de palha. Vestia uma blusa igualmente surrada, com vários botões faltando e contida na frente por um nó. Sua saia preta cobria também os joelhos e era amarrada na cintura com uma corda fina. As mãos traziam as marcas da vida difícil que levavam. Ela lamentava-se ao ver a panela no fogão de lenha com quase nada a oferecer de comida. Que vida, meu Deus!

As quatro crianças mais pareciam quatro querubins, dada a sua aparência entristecida e inocente diante de tudo que acontecia. A menor parecia ter três anos e a maior não passava de sete. Todas com os pés no chão, uma delas com um pano amarrado no pé, tentando tratar um corte feito por um galho de árvore que pisara na sua lida diária. As duas meninas, aparentando três e cinco anos, respectivamente, usavam um vestido de pano muito fino e desgastado, e os meninos mais velhos usavam apenas uma calça até os joelhos e nada mais. Todos apresentavam uma barriga proeminente e o resto do corpo sequinho. O menino maior trouxe para dentro da casa, com muito esforço para o seu tamanho, uma lata contendo água que não era muito límpida. Todos as seis pessoas sentaram-se nas banquetas e na cama e comeram o que tinham, sendo que a menor parte ficara para os pais. Após este arremedo de jantar, apagaram a lamparina e todos amontoaram-se na única cama existente. Observei tudo com muita tristeza. Como viver dessa forma seria possível? Em seguida, também adormeci, pois não tinha fome graças à reserva alimentar que recebi enquanto estava dentro do ovo.

De repente fui acordado com um grito raivoso do homem: “Mata esse barbeiro que está na menina!”

Olhei de imediato para a cama onde dormia profundamente aquela criancinha com semblante de anjo, e vi um dos meus irmãos mais velhos “grudados” no rosto da menina. Estava muito gordo, diferente de como eu o vira no dia anterior. Não entendi o que estava acontecendo, nem o que meu irmão fazia ali. Apenas vi quando a mulher o pegou na mão e o jogou na brasa do fogão de lenha. Cena medonha! Em seguida, vi o homem se aproximando do meu ninho com a lamparina acesa e começou a colocá-la nas rachaduras da parede. Fiquei apavorado e me escondi, junto com o resto dos meus irmãos, no canto mais profundo. Como não encontrasse nada o homem apagou a lamparina e começou a esbravejar: “Malditos barbeiros!”. Em seguida, todos saíram da casa sem nada comer e levando consigo as ferramentas para o árduo trabalho na roça. Eu continuava sem entender nada.

Pouco tempo depois resolvi sair da casa e me aventurar pelos arredores. Vi um monte de lenha no terreno e lá entrei. Era um lugar quente, escuro e malcheiroso. Fiquei apavorado ao ver chegar perto do lugar onde eu estava o gambá que me perseguira no dia anterior. Ele ficou farejando como se soubesse que lá havia comida. Permaneci imóvel, mas tinha a sensação de que minhas patas tremiam. Como não conseguiu nada, ele foi embora. Continuando minha exploração naquele refúgio de galhos secos, encontrei um velho barbeiro que mal conseguia caminhar e fui ter com ele. Quando me viu ficou contente, pois há muito não encontrava nenhum dos seus para conversar. Ele me perguntou de onde vinha e porque estava ali correndo perigo. Disse a ele que queria conhecer a região e que não estava entendendo o que se passava. Contei-lhe, então, o que ocorrera com meu irmão e ele me pediu para sentar ao seu lado que me explicaria.

“Há muitos e muitos anos, não havia humanos aqui e essa região era habitada por nós e muitos outros animais. Havia um mato serrado e muitas árvores com espinhos. Vivíamos em harmonia. Nós convivíamos nos ninhos de outros bichos, principalmente gambás e ratos, e lá nossos filhotes cresciam, pois tínhamos muito alimento.”

“O que nós comemos?”, perguntei curioso.

Vendo minha inocência e curiosidade, o velho barbeiro continuou: “Meu pequeno, nós só nos alimentamos de sangue. Nós chupamos o sangue dos animais e nos fartamos com isso”.

Confesso que fiquei horrorizado e, então, entendi o que meu irmão fazia no rosto daquela criança e porque estava tão gordo: ele estava se alimentando do sangue dela. Então, ele prosseguiu…

“Toda vez que nos alimentamos engordamos muito, com isto não conseguimos segurar as fezes e as eliminamos bem ali mesmo.”

“Que nojo!”, exclamei.

“Mas voltando ao nosso assunto…”, prosseguiu o velho barbeiro. “Essa nossa paz terminou. Apareceram os humanos e cortaram os arbustos e as árvores, construíram casebres e nossos amigos gambás, ratos e muitos outros fugiram assustados. Assim, fomos ficando sem comida e muitos de nós não resistiram. Outros foram em busca de novos locais e assim nos espalhamos por todo este lugar.”

“Com o tempo, fomos observando que as paredes daqueles casebres, feitas de barro, rachavam com o calor intenso. Alguns de nós, em situação de desespero por tanta fome e sem ter onde se esconder, resolveram se alojar naquelas rachaduras. Eles foram adaptando-se ao local e logo perceberam que aqueles humanos tinham sangue quente, tal qual os gambás e os ratos. Durante a noite, enquanto as pessoas dormiam, eles podiam se alimentar e voltar quase sempre em segurança para as frestas nas paredes. Às vezes, alguns demoravam mais sendo surpreendidos e, então, o destino trágico era o fogão de lenha. Mesmo assim, fomos ganhando cada vez mais espaço na região.”

Fiquei muito contente com as explicações que recebera e então peguei o caminho de volta para o meu ninho. Já estava anoitecendo e o céu pintava-se de estrelas, sem nenhuma nuvem. Chegando em casa, passei muito tempo observando aquelas pessoas. Mais uma vez alimentavam-se de um caldo ralo, que parecia não ter nada que matasse a fome. Eu continuava crescendo e ainda não sentia vontade alguma de me alimentar. Assim adormeci.

No meio da madrugada fui acordado novamente pelos gritos da mulher que esbravejava: “Malditos barbeiros !!!” O sol ainda não havia raiado e o local era iluminado pela tênue luz da lamparina. Agora, eram cinco dos meus que se fartavam do sangue das crianças. Todos eles igualmente terminaram nas brasas do fogão. Desesperado, não consegui mais dormir. Quando amanheceu, o homem, a mulher e as crianças saíram mais uma vez sem comer nada.

Pensei em sair dali e nunca mais voltar. A cena do fogão de lenha não me saia da cabeça. Olhei em volta e vi vários dos meus dormindo, redondos de tanto comer. Deram-se bem e se fartaram durante a noite, sem serem descobertos.

Fui para o terreno que ficava fora da casa à procura do meu amigo, velho barbeiro, mas não o encontrei. Perambulei por toda a área, sempre me escondendo para não ser notado. Tinha medo do que poderia aparecer na minha frente, principalmente depois das histórias que ouvira do ancião. Encontrei muitos outros animais estranhos. Alguns eram bem grandes comparados a mim e voavam alto no céu, que estava azul e sem nenhum sinal de chuva. Não gostei nada da forma como eles me olhavam e achei mais prudente entrar embaixo de umas folhas que estavam no caminho. Lá, encontrei outros dos meus que pareciam ter a minha idade ou um pouco mais, mas já com muita experiência em se aventurar por aquelas bandas. Logo ficamos amigos e conversamos bastante. Contei a eles o que ocorrera com meus irmãos e o triste fim que tiveram. Vários deles me deram conselhos:

“Quando for se alimentar, prefira as crianças, pois elas têm um sono mais pesado e geralmente dormem encostadas na parede. Dessa forma, fica mais fácil fugir para outras frestas no barro ou mesmo se esconder embaixo do colchão. Evite os adultos, pois eles podem mais facilmente acordar e nos surpreender, então, difícil escapar da raiva mortal deles.”

Eu ainda não sabia o que era sentir fome e nunca tinha me alimentado de sangue, mas ficava cada vez mais curioso a respeito.

“Mas vocês aqui fora, como fazem para se esconder e se alimentar?”, perguntei.

“Se você olhar bem, verá que neste terreno há alguns ninhos de aves, de gambás e ratos, todos eles bem escondidos. Nós moramos lá. Estamos sempre aquecidos, e durante a noite temos sangue à vontade. Só é preciso muita atenção, pois podemos fazer parte do cardápio deles. Nesses ninhos, principalmente dos gambás e dos ratos, frequentemente há filhotes e isto é uma delícia. Nós nos alimentamos fartamente e eles pouco reagem; quando chegam os pais, nos escondemos entre a palha e lá ficamos”.

Fiquei muito pensativo a respeito desse tipo de vida que me parecia mais interessante, até que um dos barbeiros comentou, em tom preocupante, que as aves, gambás e ratos estão ficando cada vez mais raros na região, como já me dissera aquele velho barbeiro.

“Eles estão fugindo para longe com medo dos humanos e nós estamos ficando cada vez mais sem opção de moradia e alimentação. É bem provável que logo tenhamos que ir para a casa em busca de abrigo e alimento, mesmo correndo o risco de terminar no fogão de lenha.”

A escuridão da noite se aproximava. Achei que era prudente voltar para casa. Despedi-me de todos e me aventurei no caminho de volta. Estava quase chegando quando fui surpreendido por uma criatura estranha e tenebrosa. Era bem maior que eu, tinha oito patas, diferente de nós que só temos seis, corpo escuro e coberto de pelos curtos. Na boca havia duas garras enormes. Era uma aranha. Fiquei aterrorizado ao vê-la e muito mais quando ela voltou-se em minha direção com um ar nada amistoso. Apenas me veio à cabeça fugir o mais rápido que minhas patas permitissem e entrar em qualquer fresta na parede que fosse pequena o suficiente para que ela não conseguisse me perseguir. Finalmente encontrei uma fenda pequena, que dava também acesso ao interior da casa. Amedrontado, fiquei lá por alguns instantes me refazendo daquele cenário macabro que acabara de enfrentar e depois me dirigi ao meu ninho, que parecia seguro.

Quando o sol já começava a aquecer o solo, acordei e percebi que os humanos estavam prestes a sair para o trabalho árduo de todos os dias. Alguma coisa diferente me chamou a atenção. O homem estava com movimentos mais lentos e sua respiração mais difícil e ruidosa; mais se apoiava na enxada que propriamente a segurava. Ainda assim, foram.

Percebi vários dos meus escondidos entre os trapos que cobriam aquela miserável cama, todos eles muito gordos e fartos. Pela primeira vez tive uma sensação estranha que não sabia o que era, mas instintivamente deduzi que estava com fome. Como era dia, não havia humanos na casa e mesmo que houvesse, não seria nada prudente me aventurar pois o fogão lá estava causando muito medo em mim. Fui então em busca de comida. Chegando ao terreno, percebi um alvoroço muito grande. Ao chegar mais perto, vi um gambá deitado junto a um arbusto. Ele estava muito quieto, respirava com dificuldade e seu corpo parecia estar muito inchado. Vários dos meus aproveitavam-se da pouca resistência do coitado e se deliciavam sugando seu sangue, mesmo percebendo que ele ficava cada vez mais fraco. Às vezes ele se mexia vagarosamente, mas não conseguia alcançar aqueles que o martirizavam. Achei tudo muito triste e, apesar da minha fome, não tive coragem de tirar proveito da situação.

Continuando minha exploração, notei no topo de um arbusto um ninho e fui verificar do que se tratava. Subi com muita calma, sempre olhando para ver se não havia nenhum perigo por perto. Para minha surpresa, lá estavam três filhotes de pássaro, bem pequenos e ainda quase sem penas.

“Será que eles têm sangue?”, refletia comigo mesmo. “Vou verificar!”

Finquei meu ferrão naquela pele fininha e tenra sem a menor dificuldade. O filhotinho sequer se moveu. Percebi então que minha ferroada era praticamente indolor. Fiquei ali por muito tempo e deliciei-me com meu primeiro repasto de sangue. Quando dei por mim, já estava anoitecendo e precisava voltar para casa. Como me havia dito o velho barbeiro, notei que minhas fezes estavam perto do local da picada. Caminhava com certa dificuldade, tamanha era minha barriga. Chegando ao meu ninho, logo adormeci e passei uma noite muito tranquila.

Pela manhã, ainda saciado pela comilança do dia anterior, olhei para o interior da casa e percebi que o homem não se sentia bem. Respirava com dificuldade, mal conseguia caminhar e suas pernas estavam muito inchadas. Não podia deitar-se e permanecia sentado na cama, com as pernas pendentes. Neste dia, apenas a mulher e as crianças saíram para a roça. Intrigado com a cena, não conseguia tirar os olhos daquele homem que parecia sofrer muito. Fiquei o dia inteiro no ninho velando aquela pobre criatura. No final da tarde, a mulher e as crianças retornaram e encontraram o homem na mesma situação. Ela preparou um mingau de fubá com um pequeno resto de carne seca, serviu boa parte para as crianças e separou um pouco para ela e seu marido, que não conseguia se alimentar pelo desconforto que sentia. Em seguida, todos adormeceram, mas o homem permanecia sentado na cama.

Ao amanhecer, estranhei que ninguém se preparava para ir ao trabalho. Ouvi a mulher dizer aos seus filhos que era domingo e que iriam à igreja assistir à missa e conversar com o padre a respeito da saúde do marido. E assim foi feito. Eu já estivera no local em uma das minhas explorações territoriais e fui para lá também.

Aos domingos e pela manhã, a comunidade da região, um total de aproximadamente 30 pessoas, reunia-se na igreja para assistir à missa e desfrutar de momentos de convívio. Compartilhavam suas dores e dificuldades, que não eram poucas, a maioria delas sem a menor perspectiva de solução. As paredes da igreja eram feitas de estacas de bambu trançadas e recheadas com o mesmo barro vermelho das casas, o que preservava o mesmo cenário de miséria, desolação e abandono. Uma cruz feita com dois galhos mais grossos estava colocada em um altar, que não tinha nada além de uma tábua sobre dois cavaletes e uma toalha clara. Os fiéis sentavam-se em bancos muito rústicos e remendados, que pareciam ruir sob o peso das pessoas. Velas acesas cercavam um pequeno vaso, no qual era possível ver alguns galhos dispostos como se fossem flores.

Fiquei sabendo que terminada a cerimônia, a mulher se aproximou do padre e explicou a situação do marido. Ao sentir a gravidade do quadro, que já vira muitas vezes em muitos dos habitantes das diversas comunidades por onde passou, ele disse que iria visitar o homem enfermo. Assim, a mulher e seus filhos voltaram para a casa, encontrando o homem no mesmo estado.

Mais tarde, o padre chegou na casa da família acompanhado por uma senhora mais idosa que era a parteira, benzedeira e a maior autoridade em saúde da região. Conversaram com o pobre homem, que se sentia muito desconfortável. Ele mal podia falar de tanto cansaço e dificuldade para respirar, além de estar com o corpo muito inchado. A mulher, com ar de conhecimento, pegou no punho do paciente e percebeu que seu coração batia de forma bastante irregular. Olhou os seus olhos, a garganta e logo fez o diagnóstico:

“É a doença do barbeiro!”, exclamou a senhora que tudo sabia a respeito das doenças da região. Com segurança, ela afirmou: “Ele precisa de um médico”.

Toda a cena me deixou muito espantado. Doença do barbeiro? Que doença é essa? Como isso é possível? Nós causamos doença em alguém? Somos tão pequeninos, como poderíamos deixar um homem daquele tamanho naquela situação?

Minha primeira reação foi de total descrédito nas suas palavras e recolhi-me ao ninho, adormecendo em seguida. Acordei durante a madrugada com muita fome. Olhei para baixo e vi a casa toda escura. O homem dormia sentado, e ali, bem juntinho da parede, aquela pequena criança em sono profundo. Desci cautelosamente, sempre preocupado que alguém acordasse e me desse o destino do fogão de lenha. Finalmente cheguei onde estava a menininha. Sua pele era tão quentinha e macia que não resisti. Comecei a me alimentar daquele sangue fresquinho e delicioso até não poder mais. Percebi que havia eliminado fezes perto do orifício da picada. Que vergonha! Saí do local rapidamente e subi pela parede com alguma dificuldade, de tão gordo que ficara. No trajeto, notei que a criança se coçava onde eu picara e espalhava minhas fezes em sua pele. Nessa noite, também dormi maravilhosamente!

O sol mal raiara e já havia movimentação na casa. O homem, acompanhado de sua mulher e do padre, saíram em busca de atendimento médico. Ouvi dizerem que o único hospital da região, no qual trabalhava apenas um médico, ficava a três horas de viagem, que seria feita em uma carroça puxada por um burro bastante velho e que andava muito devagar. Era a única maneira de ir até lá em busca de tratamento. As crianças ficaram sozinhas, sendo cuidadas pela mais velha. Neste dia não foram para a roça e puderam brincar um pouco, coisa rara de acontecer. Fiquei observando a alegria daquelas crianças que não tinham absolutamente nada, mas se divertiam com uma bola feita de pano e alguns galhos secos que faziam de conta serem bonecas. Olhando mais atentamente, percebi que elas também brincavam com um velho “barbeiro”. Elas o pegavam na mão, faziam com que ele andasse nos seus braços e até deixavam-no picar suas peles castigadas pelo sol. Gostei muito daquilo, mas não tive coragem de ir até elas. Pouco depois, passaram para outra brincadeira e deixaram o “barbeiro” em paz. Ele desapareceu em uma pilha de lenha.

Ao entardecer, o padre, a mulher e seu marido regressaram da consulta. O padre ajudou o homem a descer da carroça e com dificuldade o colocou sentado na cama. Retornou para o veículo e foi embora. Percebi na expressão da mulher um ar de tristeza e muita preocupação. Lágrimas rolavam pela sua face. Trazia consigo uma caixinha de medicamentos que lhe fora dada no hospital. Imediatamente pegou a caneca, encheu-a de água e ofereceu ao seu marido para que este tomasse o medicamento, com a esperança de melhoras. Durante a noite, ele levantou-se tantas vezes para urinar que me incomodou bastante e quase não consegui dormir. Quando acordei, todos já haviam saído e imaginei que o homem tivesse melhorado.

Saí mais uma vez para explorar a região e encontrei um velho cachorro deitado no chão. O animal não parecia nada bem. Respirava com dificuldade e estava muito inchado, mal conseguia se mover. O sol já estava muito quente, mas não parecia incomodá-lo ou talvez ele nem conseguisse reagir ao calor, dada a sua fraqueza. Novamente eu tive a sensação de fome e vi naquele animal debilitado a possibilidade de me alimentar. Dessa forma, procurei um lugar não acessível às suas patas e boca e comecei. Saciado, notei que aquele pobre animal não se mexia. Prestei atenção na sua respiração e ela foi espaçando-se cada vez mais, até cessar. Pela primeira vez, entendi o que era a morte. O que teria acontecido com aquele cão? Não entendia a situação, mas chamou-me a atenção a dificuldade em respirar, semelhante a do homem na casa. Seria também a “doença do barbeiro”, como afirmara anteriormente a velha senhora? Preocupado, voltei para o meu ninho. Mais tarde chegaram as crianças, a mulher e o marido. Observei que o homem estava menos ofegante, mas ainda com as pernas muito inchadas. Com muita dificuldade, não podia deixar de ir para o trabalho árduo da roça. Ele sentou-se na cama, enquanto as crianças brincavam um pouco no terreno e a mulher preparava alguma coisa para comer. Percebi que ela trouxera uma ave morta e começou a despedaçá-la, colocando-a em seguida na panela com água que fervia no fogão. Pouco depois, comeram, e como já escurecera, acomodaram-se na cama para dormir. O homem permanecia sentado. Dormi preocupado. A cena do pobre cachorro não me saía da cabeça, nem daquela ave que fora devorada sem dó.

Quando acordei todos já haviam saído. Eu me sentia estranho, como se aquela comilança do dia anterior não me tivesse feito bem. Não sabia exatamente o que era, mas percebi que havia algo de estranho comigo. Fiquei o dia inteiro recolhido no ninho. Ao final da tarde, quase escurecendo, todos retornaram e não gostei nada da aparência do homem. Parecia muito mais cansado e ofegante, muito inchado até na barriga, e com dificuldade até para abotoar a sua surrada camisa, que permanecia completamente aberta. Essa noite ele não quis se alimentar e foi se recostar na cama, sentado com as pernas para fora, adormecendo.

Os dias foram passando, mas aquele cenário monótono foi interrompido por um fato pavoroso. O homem, que estava em pé ao lado da porta, subitamente caiu no chão desacordado. Estava sozinho na casa, pois sua mulher fora lavar uns trapos em uma bacia que ficava fora da casa e as crianças estavam catando lenha para ser usada no fogão. Subitamente ele se levantou como se nada tivesse acontecido. Quando todos retornaram, o homem permaneceu calado e não comentou o ocorrido. Esse fato ocorreu outras vezes, e ele sempre estava só. Por não querer preocupar a todos, nada contou a respeito.

Numa outra manhã de calor intenso, notei que aquela criancinha que tanto me servia de alimento estava estranha. Não queria sair da cama, aparentava cansaço e notei que seu olho direito estava inchado. Logo me lembrei da velha senhora e de suas palavras… “É a doença do barbeiro.” Confesso que fiquei apavorado e senti culpa, sem saber ao certo como isto acontecera. Tantas outras vezes me alimentei de seu sangue após ter sugado aquele pobre cachorro moribundo e só agora causei doença nessa criança? Como era possível? O que teria acontecido? Seria eu o culpado?

Os dias foram passando e eu notava a criança cada vez mais enfraquecida. Não brincava e mal se alimentava por estar muito cansada. O quadro durou por várias semanas, até que ela começou a melhorar, voltando ao seu estado normal. Sua recuperação me deixou aliviado, pois imaginava não ter causado nenhum mal àquele anjinho. Para meu espanto, todas as outras crianças, menos uma, apresentaram quadro semelhante. Eu não havia me alimentado de nenhuma delas, mas os meus irmãos, sim.

Um belo dia, andando pelas redondezas, encontrei aquele velho barbeiro a quem não via há muito tempo e que esclarecera todas minhas dúvidas. Perguntei, então, a respeito do que acontecera com aquelas crianças. O que ele me disse foi aterrorizante. Seguramente eu me contaminara com o sangue daquele cachorro doente e por toda minha vida iria contaminar aqueles de quem me alimentasse. Fiquei arrasado e prometi a mim mesmo nunca mais me alimentar de ninguém daquela casa. E assim foi feito… quando sentia fome, procurava nas redondezas um ninho de ave com filhotes ou um velho gambá que mal se mexesse. Nunca mais me alimentei do sangue de ninguém daquela família. O remorso me doía bastante.

Do meu ninho podia ver aquele homem cada vez mais cansado e emagrecido, mas que não deixava de trabalhar, mesmo que a duras penas. O cenário era a cada dia mais triste e sem esperanças. A comida e a água cada dia mais escassas. A mobília da casa desmoronando progressivamente. As crianças crescendo sem perspectivas. Que situação horrível, meu Deus!

Um belo dia, uma notícia animadora: chegara ao vilarejo uma jovem professora, que iria assumir a escola em ruinas do lugar e possibilitar a quem quisesse, principalmente às crianças, aprender a ler e a escrever, para finalmente entender os amontoados de letras dispostas naquelas folhas de jornais velhos, que serviam de tapetes. Pensei comigo: “Será esta uma fagulha de alegria?”

Nos dias que se seguiram houve uma grande mudança no comportamento das crianças. Três vezes por semana, bem cedinho, elas iam alegres para a escola e lá ficavam até por volta das onze horas, quando voltavam para casa. Nesses dias, participavam dos trabalhos na roça apenas no período da tarde. Eu me admirava vendo-as tentando ensinar aos seus pais como juntar aquelas letras e formar palavras; porém, a dificuldade deles era muito grande. Elas já esboçavam a leitura dos pedações de jornal que ficavam no chão e isto lhes proporcionava muita alegria.

Os dias se passavam e voltei a notar que o homem apresentava desmaios mais frequentes, agora presenciados por todos na casa. Após estes episódios, ele levantava como se nada tivesse acontecido. Isso me incomodava muito, pois ele permanecia cansado e muito inchado. A imagem do cachorro morrendo não saia da minha mente, e a atual situação do homem parecia semelhante. A esposa, cada dia mais tensa, não queria demonstrar sua preocupação para as crianças, que não notavam a sensível piora na saúde do pai.

Certo dia, acordei antes de o sol nascer, preocupado com todo o cenário do dia anterior. Em seguida, a mulher levantou-se, juntamente com as crianças, mas o homem permanecia imóvel. A mulher tentou acordá-lo, sacudiu-o, mas ele não se movia. A cena seguinte foi muito dolorosa: ela gritava e chorava desesperadamente. As crianças também estavam aos prantos e gritos, e abraçavam o pai. Ele estava morto. Morrera dormindo como poucos merecem fazê-lo. O desespero era apavorante. Ninguém saía de perto do homem deitado na cama com os pés pendentes para o chão. O que seria agora daquela mulher e daquelas crianças?

Depois de muito tempo vivendo aquela cena imóvel, a mãe mandou a criança mais velha chamar o padre. Ela ainda chorava desesperadamente, mas ainda assim acatou o pedido da mulher e saiu em busca do religioso, chorando de dar dó.

Finalmente, o padre chegou àquela casa e constatou a morte do pobre homem. Fez orações, desejando paz à sua alma, e tentou confortar a mulher e as crianças, mas faltavam argumentos diante de tanta miséria.

Ao pobre homem, restava apenas um sepultamento na própria terra em que sempre vivera. A mulher e as crianças deveriam prosseguir com suas miseráveis vidas, agora sem o marido e pai.

Os dias foram passando numa monotonia angustiante, até que chegou na cidade um grupo de homens que se propunham matar todos os barbeiros da região. Eu e meus irmãos, todos já adultos, ficamos apreensivos com a notícia, tentando imaginar uma estratégia de defesa. Deveríamos sair da casa e rumar para o terreno, igualmente perigoso?

Pessoalmente, decidir ficar, já que, por pura covardia, não queria enfrentar os perigos dos gambás, aranhas e cachorros sem ter um porto seguro para retornar. Lembrava-me de quando o homem com sua lamparina fumegante nos procurava entre as frestas e eu me escondia bem lá no fundo, o que me parecia mais seguro.

Certo dia, chegaram na casa dois homens vestindo roupas estranhas, ambos com uma máscara no rosto e que lhes dava um aspecto amedrontador. Eles pediram para a mulher e as crianças saírem da casa e só retornarem depois de algumas horas. Fiquei curioso quando começaram a misturar líquidos em uma lata. O cheiro estava insuportável. Logo pensei em fugir, mas era tarde demais. Escondi-me o mais fundo possível naquela fresta.

“*O que está acontecendo*?”, perguntei-me. Eles vinham em minha direção, espirrando aquele líquido que penetrava fundo no meu ninho, deixando a mim e aos meus irmãos totalmente molhados. De repente, percebi meus irmãos caindo um a um no chão, imóveis. Poucos como eu restavam. Sentia-me tonto, fraco e com muita dificuldade de me mover. Permaneci quieto.

Os homens estranhos foram embora e algumas horas depois a mulher e as crianças voltaram, recolheram vários barbeiros mortos do chão e os jogaram no fogão à lenha. Triste fim…

O cheiro irritante diminuiu progressivamente até o ambiente voltar ao normal. Por que vários dos meus morreram, e eu não? Será que tivera uma nova chance para não cometer mais o erro de causar a “doença do barbeiro” naquelas pessoas tão sofridas?

Quase um ano se passara desde que saí daquele ovo. Sinto-me muito fraco, com dificuldade para caminhar e sem conseguir forças para sair e me alimentar. Meus olhos estão turvos e tudo está bastante escuro. Estou com muito sono e sinto que vou adormecer.

Revendo minha trajetória, entendo que todo o mal que causei não foi intencional, mas apenas pela sobrevivência. Tomara que isso possa ser corrigido um dia. “Não consigo mais respirar…”

“Ao culparmos o barbeiro pela disseminação dessa terrível doença, temos que analisar com imparcialidade o papel dos humanos na história. Ao tentar sobreviver, muitas vezes tomamos atitudes impensadas que podem prejudicar não somente a nós. ”
