# Supplementary material for: A Doença de Chagas: Seria o Barbeiro o Único Culpado?
Source: Arq Bras Cardiol. 2021 Jul 14;117(1 Supl 1):1–7. [Article in Portuguese] doi: 10.36660/abc.20210290 (PMC8291899; doi:10.36660/abc.20210290)
Supplement: Translation

# Translation: Chagas Disease: Are Kissing Bugs the Only Culprit?

**Authors:** Abilio Augusto Fragata

**Affiliations:** São PauloSP Brazil Instituto Dante Pazzanese de Cardiologia - Laboratório de Doença de Chagas, São Paulo, SP – Brazil

**Keywords:** Chagas Disease/history, Trypanosoma Cruzi, Chagas Cardiomyopathy, Myocardites, Mortality

## Preface

Chagas disease, first described by Carlos Chagas in 1909, is caused by a single-celled parasite (*Trypanosoma cruzi*). Throughout history, the main mechanism of transmission has been via contact with the feces of an insect infected with the parasite. This insect, known as the “kissing bug” in English, is popularly known as “*barbeiro*,” “*chupão*,” or “*chupança*” in Brazil. The “kissing bugs” are not born with the parasite in their intestine; rather, they acquire it by feeding on the blood of infected mammals. (This disease only affects mammals). When taking a blood meal, the insects eliminate feces, where the parasites are present, and the parasites can contaminate the bite, allowing the parasite to enter the organism. In this manner, the disease begins. The kissing bugs mainly make their nests in cracks in clay walls of houses made in a style known as “*pau-a-pique*” in Brazil. These houses are very common in diverse regions of Brazil, especially throughout the *sertão* of the Northeast Region. Other mechanisms of transmission can occur, for example, via blood transfusion, organ donation, mother-to-child transmission, ingestion of contaminated foods, etc.

During the acute phase of the disease, complaints may be mild or even non-existent. They may include general manifestations, such as low fever, malaise or more severe ones, such as shortness of breath and swelling.^1^ As bites from kissing bugs usually occur on the face, swelling of the eyebrows, known as the Romaña sign, may occur. As the years pass, generally after 10 years or more, cardiac complaints appear in one third of patients, such as fatigue, shortness of breath, and swelling in the body (heart failure).^2^ In some cases, the electrical system of the heart may be compromised, with alterations in cardiac rhythm, fainting, and even sudden death. Dilatation of the esophagus and the colon can also occur, with progressive difficulty in swallowing food and defecating.^1^

Since the first issues, which date back to 1948, the *Brazilian Archives of Cardiology* have registered advances in medical knowledge regarding this specialty. Even with few technical resources, diverse researchers of that time showed brilliant clinical reasoning. The first 10 articles on Chagas disease in this journal date from 1948 to 1958.^3^^-^^12^

At the end of the 1940s, the figures included in the articles, such as those published in 1948 and 1949 (Figure 1), already reflected groundbreaking didactic interest on the part of the *Brazilian Archives of Cardiology*.

**Figure 1:** (A) Left, heart of case 26; right, normal heart. On radioscopy: large myogenic heart, almost completely stopped. (B) Parasite cluster in the cardiac fiber (S. cruzi).^3^^,^^5^

## My story

When we were still eggs, my mother left my siblings and me in a dark place that smelled like clay. Lucky for us. When I came out of the egg, I noticed that many of my siblings had already come out, but others weren’t reacting. Later, I would understand that they would never come out. I don’t know who my mother is, or my father either.

When I was still little, exploring the territory where I was born, I saw that my nest was in a small crack in a very strange house. It was made with interlaced sticks of bamboo that were filled in with red clay. There was a peculiar smell, a mixture of sweat and burnt wood. There were several very old objects. A little lamp that was turned off, on top of a wooden shelf that was nearly rotten close to the door, which was wide open and made of pieces of planks, with lots of cracks between them. There was a wood-burning stove with embers on top and a pot that was almost empty, just boiling water and a few pieces of manioc. Next to the wall, right below my nest, I could see a double bed that was patched up around the feet and the head, on the verge of collapsing. On top, there was a threadbare mattress with a blanket that was also in tatters, with clumps of straw sticking out of the parts that were torn. On the other wall, an open window with a table beneath it, also worn down, with all four legs on the verge of falling apart. There were no towels, just two empty pea tins that were used as drinking cups. Next to them, two plates that were chipped all over with a worn-out spoon on top of each. The floor was made completely of raw earth, with pieces of wood and leftover food, as well as splattered contents from the wood-burning stove. The roof was made entirely of dry straw with a lot of holes that let sunbeams in. There was no one home. Looking at this scenario, I though that my nest was pretty comfortable by comparison.

Several of my siblings had already gone and I didn’t hear from them anymore. The ones who were there with me and I found the environment very sad. We didn’t even know who we were. I would later learn that we were insects known as “kissing bugs.”

Leaving my nest, I started to explore the house and I went out the window, afraid of what I might find there. The sun was blazing and there was not even a little breeze to cool us off. The earth was covered with red clay, and we rarely saw patches of brush with yellow leaves that were twisted by the heat. I thought that the inhabitants must not feel thirst, because I couldn’t see any traces of water. A little later I heard a noise and I saw an emaciated dog with its tongue hanging out lazily slinking along the burning ground. I noticed that it scarcely had the strength to walk and bark at the same time. It came close and, in fear, I hid in a crack that I found on the outside of that little hut. I stayed still there while the dog sniffed incessantly, as though it were hunting something to eat. A little while later it left, went into the house, and lay down on the ground next to the door, sleeping heavily, perhaps to take its mind off of how hungry it felt. Continuing my exploration, I saw another animal. It was very strange, and I later learned that it was called a skunk. When it saw me, it immediately ran toward me with a horrifying expression, demonstrating the intent to, at my expense, sate its hunger. Once again, I hid in the strategic haven in the crack in the wall, which also provided an opening that went inside the house. As it was already nighttime, I decided to go back to the nest where I would be safe. I admit that I fell asleep. I don’t know how long. I woke startled by a loud noise inside the house. It was very dark, and I couldn’t see anything. Then, a faint light came on and I saw that the lamp had been lit. Imagine how frightened I was when I saw a man, a woman, and four children speaking loudly inside the house. The man was short and very lean, and the skin of his face was wrinkled, showing signs of suffering from constant exposure to the sun. He wore busted up sandals on his feet. His pants hardly covered his ankles, and they were all patched up, with several tears that couldn’t take another stitch. His hands, which were calloused from the daily toil on the small farm, were incapable of finer movements, and they handled everything with brutality. He was smoking a straw cigarette that spread a foul-smelling smoke, but still seemed comforting to him.

The woman wore a resigned expression, and she was equally thin. She walked barefoot, and her feet were calloused. On her head, she wore a cloth that must have been white one day, but was yellow by then. The cloth hid almost all of her dark hair and the locks that weren’t covered looked like straw. She wore a blouse that was similarly in rags. It was missing several buttons and it was held closed by a knot in the front. Her black skirt also covered her knees, and it was tied around her waist with a thin rope. Her hands bore the signs of the difficult life she led. She lamented when she saw the pot on the wood-burning stove with so little to offer to eat. What a life, my God.

The four children seemed more like four cherubim, given their sad and innocent appearance in view of everything that was going on. The youngest seemed to be around three, and the oldest wasn’t older than seven. All of them walking barefoot. One of them had a rag tied around her foot, in the attempt to treat a cut made by a branch that she had stepped on in her daily struggle. The two girls, who appeared to be three and five, were wearing dresses made of very thin cloth that was worn-out, and the boys, who were older, were wearing pants that reached their knees and nothing else. All of them had stomachs sticking out, and the rest of their bodies were skeletal. The oldest boy, with a great deal of effort for his age, dragged a tin container of water, which was not very clean, into the house. All six sat around the table or on the bed, and they ate what there was to eat, the largest portions being for their parents. After this paltry dinner, they turned out the lamp, and all six piled into the only bed they had. I observed all of this with great sadness. How was it possible to live like that? Shortly thereafter I fell asleep too, because I wasn’t hungry thanks to the food reserve I had received while I was inside the egg.

All of a sudden, I woke to angry shouts from the man, “Kill that kissing bug that's on the girl…”

Instantly, I looked at the bed where that little child was fast asleep with the expression of an angel and I saw one of my older siblings “stuck” on the little girl's face. He was very fat, in contrast to how I’d seen him the day before. I couldn’t grasp what was going on or what my brother was up to there. The only thing I saw was the woman who grabbed him and threw him in the embers of the wood-burning stove. Terrifying scene. Then I saw the man come close to my nest with the lamp on and he started putting it in the cracks in the wall. I was afraid, and I hid, along with the rest of my siblings, in the far corner. Not finding anything, the man turned off the lamp and started swearing, “Darn kissing bugs.” Shortly thereafter, they all left the house without having anything to eat, taking their tools with them to the backbreaking work on the farm. I stayed there, trying to get a grip.

A little while later, I decided to leave the house and wander around the surrounding area. I saw a pile of wood outside and I went in. It was a dark, warm place with a bad smell. I was frightened when I saw that skunk, the one that had chased me the day before, coming close. He started smelling around as if he knew there was food there. I stayed still, but I had the feeling my legs were shaking. When he didn’t find anything, he went away. Continuing my exploration of that hiding place full of dry sticks, I found an old kissing bug who could scarcely walk, and I went over to him. He was glad to see me because it had been a long time since he had met one of his own to talk to. He asked me where I came from and why I was putting myself in harm's way. I told him that I wanted to get to know the region and that I couldn’t grasp what was going on around me. Then I told him what had happened to my brother and he asked me to sit by his side so he could explain.

“Many years ago, there were no humans here and this region was inhabited by us and many other animals. There was a thick scrubland and lots of trees with thorns. We lived in harmony. We made our houses in other animals’ nests, mostly skunks and rats, and that's where our children grew up, because we had a lot of food.”

“What do we eat?” I asked curiously.

Seeing my innocence and curiosity, the old kissing bug went on, “Young one, we only eat blood. We suck blood from other animals and we are satisfied with that.”

I confess that I was horrified, and then I understood what my brother was doing on that child's face and why he was so fat; he was feeding on her blood. He went on.

“Every time we eat, we get very fat and then we can’t keep our feces in, and we end up defecating right there.”

“That's gross,” I exclaimed.

“Back to what I was saying,” the old kissing bug went on. “Our peace came to an end. Human beings arrived, and they cut down the bushes and trees. They made little huts, and our friends the skunks and rats and many others fled in fear. So many of us had nothing to eat and some of us died out. Others went looking for new places, and that's how we spread throughout this place.”

“As time went on, we learned that the walls of those houses, made of clay, cracked in the intense heat. Some of us, in despair, out of hunger and lack of a place to hide, made up our minds to make a home in those cracks. They got used to the place and started to notice that those humans also had warm blood, just like the skunks and rats. At night, while they sleep, kissing bugs can eat and almost always get back safely to the cracks in the walls. Sometimes they take too long and get caught and end up in the wood-burning stove. All the same, we started expanding throughout the region.”

I was satisfied with the explanation he had given me, so I started back to my nest. It was already getting dark and the sky was bright with stars, without any clouds. When I got home I spent a long time observing those people. Once again they ate a measly soup that didn’t seem to have much to satisfy their hunger. I kept growing, but I still didn’t feel the need to eat. So I fell asleep.

In the middle of the night, I was awoken again by the woman who was yelling, “Darn kissing bugs!” The sun had not come up and the house was already lit by the faint light of the lamp. Now there were five of my siblings who were sucking the children's blood. All five of them ended up in the embers of the wood-burning stove. In despair, I couldn’t fall asleep. When the sun came up, the man, the woman and the children left again without eating anything.

I thought about leaving there and never coming back. I couldn’t get the scene with the wood-burning stove out of my head. I looked around and saw several of my siblings sleeping, fat from eating so much. They had done a good job of eating at night without being discovered.

I went outside looking for my friend, the old kissing bug, but I couldn’t find him. I roamed around the perimeter, always hiding so as not to be noticed. I was afraid of what I might find, especially after the old man's stories. I came across a lot of strange animals. Some of them were rather large compared to me and they were flying high up in the sky, which was blue and showed no signs of rain. I didn’t like the way they looked at me at all, and I thought it would be wise to stay beneath the leaves in the path. There I found many of my own kind who seemed to be my age or a bit older, but they already had more experience, and they were roving around those parts. We quickly became friends, and we talked a lot. I told them what had happened with my siblings and how they met their tragic end.

Many of them gave me advice.

“When you eat, go for the children, because they sleep more heavily and they generally sleep up against the wall. That way, it is easier to run away inside other openings in the clay or even to hide beneath the mattress. Avoid the adults, because they wake up more easily and catch us, and it's hard to get away from them when they are in a deadly rage.”

I still didn’t know what it meant to feel hungry and I had never had a blood meal, but I was getting more and more curious about it.

“But out here, how do you hide and find food?” I asked.

“If you look around, you’ll see that, in this field, there are a lot of nests that belong to birds, skunks, and rats, all very well hidden. We live there. We’re always warm and at night we have all the blood we want. We just need to pay close attention, because we can end up on their menu. In those nests, especially the ones that belong to the skunks and the rats, there are usually babies and they are delicious. We can eat all we want, and they hardly react; when it comes to the parents, we hide between the straw and that's where we stay.”

I thought hard about this type of life, which seemed more interesting to me, and some of the kissing bugs even commented in a worried tone of voice that these birds, skunks, and rats were getting rarer and rarer around the region, just like that old kissing bug had already told me.

“They are running far away, afraid of human beings, and we have less and less choice of where to live and what to eat. It's very likely that soon we’ll have to go inside the house to look for food and shelter, even if we have to run the risk of the wood-burning stove.”

It was getting dark. I thought it wise to get home. I said goodbye to my new friends and set off on the way home. I was almost there when I was startled by a dark, strange creature. It was much larger than me. It had eight legs, in contrast to us, who only had six. Its body was dark and covered with short hair. It had two giant fangs in its mouth. It was a spider. I was terrified when I saw it and even more so when it turned toward me in a way that wasn’t friendly at all. The only thing that went through my mind was to run away as fast as I my feet would let me and go into the first crack in the wall that was small enough for the spider not to be able to come in after me. Finally, I found a little crack that also extended into the house. I stayed there for a few moments in fear, gathering my thoughts after that frightful scene that I’d just faced, and then I went toward my nest, which seemed safe to me.

When the sun was already starting to heat up the ground, I woke up and I noticed that the humans were getting ready to head out toward their daily toil. Something there drew my attention. The man was moving more slowly and his breathing was laborious and noisy, but he leaned on his shovel, which gave him some support. But they went off.

I noticed several of my own kind between the rags that covered that pitiable bed, and they were all fat and satisfied. For the first time I had a strange feeling. I didn’t know what it was, but instinctively I understood that I was hungry. Since it was daytime, there were no human beings in the house, and, even if there were, it would not have been wise on my part to wander out because the stove was there, and it was frightening. So I went looking for food. When I got outside I noticed a great deal of commotion. When I got closer, I saw a skunk lying down next to a bush. It was very quiet. It was having difficulty breathing and its body seemed very swollen. Several of my own kind were taking advantage of the poor animal's lack of resistance and they were sucking its blood, even though they saw that it was getting weaker and weaker. Sometimes it moved slowly, but it wasn’t able to reach the animals that were causing it so much suffering. I thought it was very sad and even though I was hungry I couldn’t bring myself to take advantage of the situation.

Continuing my exploration I noticed a nest on top of a bush, and I went to see what it was. I climbed very slowly, keeping my eyes open to see if there was any danger nearby. To my surprise there were three baby birds. They were very small, and they have almost no feathers.

“Might they have any blood?” I asked myself. “I’m going to check.”

I was able to put my sucker in the skin that was thin and soft, without any difficulty. The baby bird scarcely moved. That was when I noticed that, when I sucked their blood, it was practically painless. I stayed there for a long time enjoying my first blood meal. When I came to myself, it was already getting dark, and it was time for me to go home. Like the old kissing bug had told me, I noticed that my feces were close to the place where I had bitten it. I walked with a certain difficulty on account of the size of my stomach. When I got back to my nest, I fell asleep and had a very peaceful night.

In the morning, still satiated from eating so much the day before, I looked around the inside of the house and noticed that the man did not feel well. He was having difficulty breathing. He could scarcely walk, and his legs were very swollen. He wasn’t able to lie down, and he remained seated in the bed, dangling his legs. Just that day, the woman and the children went out to work on the farm. Fascinated by the scene, I couldn’t take my eyes off that man who seemed to be suffering a lot. I spent the whole day in my nest watching that poor creature. At the end of the afternoon, the woman and the children came back and found the man in the same situation. She prepared corn mush with what little was left or their dried meat, gave a good part of it to the children and set some aside for herself and her husband, but he wasn’t able to eat on account of how uncomfortable he was. So they all went to sleep, but the man remained seated in the bed.

When it was morning, I found it strange that no one was getting ready for work, and I heard the woman telling her children that it was Sunday and that they would go to church for mass and talk to the priest about her husband's health. And then they went away. I had already been there on one of my excursions around the territory and I went there too.

Sunday mornings, the whole community, which was around thirty people, got together in the church for mass and to spend time together. They shared their difficulties and concerns, which were numerous, and often without any prospective solution. The walls of the church were made of interweaved bamboo sticks that were filled in with the same red clay that the houses were made of, maintaining the same setting of misery, desolation, and abandonment. A cross made of two thicker branches was placed on top of an altar made of nothing more than a plank on top of two wooden stands and a white cloth. The worshippers sat on very rustic benches that were patched together and that seemed like they were about to collapse under the weight of the people. Lit candles surrounded a small vase where we could see a few branches arranged as though they were flowers.

I found out that, when the ceremony was over, the woman went up to the priest and explained her husband's situation to the priest. When the priest heard about the severity of the situation, which he had already seen many times in many of the inhabitants of the diverse communities he had visited, he said that he would visit the sick man. So, the woman and her children went home where they found the man in the same state.

Later, the priest arrived at the family's home, accompanied by an elderly woman, who was the midwife, blessing-giver, and the region's leading authority on health. They talked with the poor man who felt so uncomfortable. He could hardly speak on account of how exhausted he was and his difficulty breathing, and his body was very swollen. The woman, with a knowing air, took hold of the patient's wrist and realized that his heart was beating in a very irregular manner. She looked at his eyes and throat and quickly made her diagnosis.

“It's the kissing bug disease,” exclaimed the elderly woman who knew everything about diseases in the region and she stated with certainty, “He needs a doctor.”

That scared me very much. Kissing bug disease? What type of disease is that? How was it possible? Could we cause someone sickness? We are so small. How could we put a man that big in such a situation?

My first reaction was complete disbelief in her words, and I curled up in my nest and soon fell asleep. I woke up in the middle of the night, and I was very hungry. I looked down and saw that the house was totally dark. The man had fallen asleep sitting up, and there, up against the wall, was a little child fast asleep.

I went down carefully, constantly worried that someone might wake up and throw me in the stove. Finally, I reached the little girl. Her skin was so warn and soft that I couldn’t help myself. I started eating her blood, which was fresh and delicious, until I couldn’t eat anymore. I noticed that I had eliminated feces close to the bite wound. How embarrassing. I left the place as quickly as I could and climbed the wall with some difficulty on account of how fat I had gotten. On the way, I noticed that the child was scratching where I had bitten her, and she was spreading my feces across her skin. That night, I also slept very soundly.

As soon as the sun came up, they were already moving about the house. The man, accompanied by his wife and the priest, were going to seek medical attention. I heard they say that the only hospital in the region, where there was only one physician, was three hours away and they were going on a cart drawn by an old donkey that went very slowly. That was the only way to go look for medical treatment. The children stayed there alone, and the oldest one took care of them. That day, they didn’t work on the farm, and they could play a little bit, which was a rare sight to see. I stayed there observing how happy those children were. They had absolutely nothing, but they had fun with a ball made of rags and some dry sticks that they pretended were dolls. I watched closely and I realized that they were also playing with an old “kissing bug.” They were picking it up, letting it walk on their arms and even letting it bite their skin, which was damaged by the sun. I really liked that, but I didn’t have the courage to go over to them. A little while later, they started playing something else and they left the “kissing bug” alone. It disappeared into the woodpile.

In the evening, the priest, the wife, and the husband came back from the physician. The priest helped the man get out of the cart and, with difficulty, helped him sit down in the bed. He went back to the cart and left. I realized that the woman had a sad and very concerned expression. Tears were rolling down her face. She was carrying a little box with a medication that she had received at the hospital. She immediately got the cup, filled it with water, and gave it to her husband so that he could take the medication, in the hope that he would get better. That night, he got up to urinate several times, and it bothered me so much I almost couldn’t get to sleep. When I woke up, everyone had already left, and I imagined that the man had gotten better.

Once again, I went out to explore the region. I found an old dog lying down on the ground. It didn’t seem well at all. It was having difficulty breathing, and it was so swollen it could hardly move. The sun was already very hot, but it didn’t seem to bother the dog, or maybe it couldn’t even react to the heat, given how weak it was. Again, I felt hungry and I saw, in that debilitated animal, a chance to eat. So I found a place its feet and mouth couldn’t reach and started eating. When I was full, I realized that that poor animal wasn’t moving. I paid attention to its breathing, and it was getting slower and slower, until it stopped. For the first time, I understood what death was. What might have happened to that dog? I couldn’t understand that, but what drew my attention was the difficulty breathing that resembled the man in the house. Could it also be the “kissing bug disease,” like the elderly woman had said before? Worried, I went back to my nest. Later the children, the woman, and the man came back. I noticed that the man was panting less, but his legs were still very swollen. Difficult as it was, he didn’t stop going out to work on the farm. He sat on the bed while the children played a bit outside and the woman prepared something to eat. I noticed that she was carrying a dead bird. She started to break it into pieces, and then she put it in a pot with boiling water on the stove. A little later, they ate, and, as it had already gotten dark, they got comfortable in the bed to sleep. The man remained seated. I fell asleep worried. I couldn’t get the scene with that poor dog out of my mind, or the one of that bird that had been so mercilessly devoured.

When I woke up everyone had already gone out. I felt strange, as though the feast from the day before wasn’t good for me. I didn’t know exactly what it was, but I realized that something strange was happening to me. I spend the whole day in the nest. At the end of the afternoon, when it was already getting dark, they all came back, and I didn’t like the way the man looked. He looked very tired, and he was panting. He was still very swollen, even in his stomach, and he was having difficulty buttoning his torn shirt, which he left totally open. That evening, he didn’t feel like eating and he went to relax in bed, sitting with his legs dangling, nodding off.

The days passed, but that monotonous scenario was interrupted by a frightening event. Standing up next to the door, the man suddenly fell unconscious on the ground. He was alone at home, because his wife had gone to wash some rags in a tub outside the house, and the children were gathering wood for the stove. All of a sudden, he stood up as if nothing had happened. When they all came home, the man stayed quiet and he didn’t say anything about what had happened. This happened other times, and he was always alone. So as not to worry the others, he didn’t say anything about it.

Another hot day, I realized that the little child who had provided me with so much food also seemed strange. She didn’t want to get out of bed, she seemed tired, and I noticed that her right eye was swollen. I remembered the elderly woman and her words, “It's the kissing bug disease.” I confess that I felt scared and guilty, without knowing exactly how that had happened. On many other occasions, I had fed on her blood after having bitten that poor dying dog, and had I, just now, given this child that disease? How could that be possible? What might have happened? Could I be the one to blame?

The days went on and I noticed that that child was getting weaker and weaker. She didn’t play. She scarcely ate because of how tired she was. That went on for several weeks until she started getting better, going back to normal. Her recovery was a relief to me, because I imagined that I hadn’t hurt that little angel after all. To my fright, all the other children, except for one, starting showing the same symptoms. I hadn’t fed off of any of them, but my siblings had.

One day, walking around the area, I met that old kissing bug, who I hadn’t seen for a long time, who had answered all my questions before. So I asked him about what had happened to those children. What he told me was terrifying. I had certainly become contaminated by the blood of that sick dog and for the rest of my life I would contaminate those I fed from. I was shocked, and I promised myself that I would never bite anyone from that household again. And so it was… When I felt hungry, I looked around for a bird's nest with baby birds or an old skunk that was hardly moving. I never fed from anyone in that family again. The remorse hurt too much.

From my nest, I could see that man getting more tired and thinner every day, but he never stopped working, even when it was painful. The scenario was sadder and more hopeless every day. Food and water were scarcer and scarcer. The furniture around the house progressively falling apart. The children growing up without prospective. What a horrible situation, my God!

One day, a piece of promising news: A young teacher had arrived in the little village, and she was going to take charge of that old school that was in ruins and give an opportunity to anyone, especially to the children, who wanted to learn to read and write, and then they would be able to understand all those letters arranged on those old newspapers they used as rugs. I thought to myself, “Could this be a spark of hope?”

The following days, the children's behavior changed. Three times a week, early in the morning, they went to school happy, and they stayed there until around eleven in the morning, when they came home. Those days, they only worked on the farm in the afternoon. I sat there impressed, watching them teach their parents how to put together letters to form words, but it was very difficult for them. They were already trying to read the big pieces of newspaper on the floor, and that made them very happy.

The days went by, and I started noticing that the man was fainting more often. Now everyone in the house witnessed it. After the episodes, he would stand up as if nothing had happened. That bothered me a lot, because he seemed tired and very swollen. I couldn’t get the image of that dying dog out of my head, and the man's situation seemed very similar. His wife, getting more worried by the day, didn’t want the children to see how concerned she was. They didn’t notice the slight worsening of their father's condition.

One day, I woke up before the sun came up, worried about the whole scenario from the preceding days. Shortly thereafter, the woman got up, together with the children, but the man didn’t move. The woman tried to wake him up, shook him, but he didn’t move. The scene was very painful. The woman yelling and crying desperately. The children crying and hugging their father. He was dead. He died in his sleep, like few people deserve. Their despair was terrifying. No one left his side as he lay there in bed with his feet dangling over the ground. What would become of that woman and those children now?

After that motionless scene continued for some time, the mother told the oldest child to go and call the priest. The child was still crying in despair, but he obeyed his mother and went looking for the priest, crying in a way that hurt to see.

Finally, the priest arrived at that house and confirmed that the man had died. He prayed for his soul to rest in peace and tried to comfort the woman and the children, but there was little to say in the face of so much misery.

The only thing left for that man was to be buried in the land where he had always lived. The woman and the children would have to continue their tragic lives, without a husband and a father.

Day after day went by, in an agonizing monotony, until a group of men came to town, proposing to kill all the kissing bugs in the region. My siblings and I, all adults by then, were very apprehensive about this news, trying to imagine a defensive strategy. Should we leave the house and look for an equally dangerous area?

I personally decided to stay, because, out of cowardice, I didn’t want to face the dangers of skunks, spiders, and dogs, without having a safe haven to return to. I remembered when the man with his smoking lamp would look for us between cracks in the wall, and I would hide way in the back. That seemed safer to me.

One day, two men came to the house wearing strange clothes, with masks on their faces that made them look scary. They asked the woman and the children to leave the house and come back a few hours later. I was curious when they started mixing liquids in a tin container. The smell was unbearable.

Then I started thinking of running away, but it was too late. I hid as far as possible in that crack.

“What's happening?” They were coming toward me spraying that liquid that went back into my nest, soaking my siblings and me completely.

All of a sudden, I saw my siblings fall on the ground without moving. Few of us were left. I felt dizzy and weak, and I could hardly move. I stayed still.

The strange men went away and a few hours later the woman and the children came back, gathered many dead kissing bugs from the ground and threw them in the stove. Sad end.

That noxious smell decreased progressively, until the environment went back to normal. Why had so many of my own kind died and I hadn’t? Was it possible that I had been given a second chance not to make the same mistakes that caused the “kissing bug disease” in those people who suffered so much?

A year had already gone by since I had left that egg. I feel very weak. It is hard to walk, and I don’t have the strength to go out and find food. My vision is blurry and everything is very dark. I feel very sleepy and I am going to fall asleep.

Looking back on my trajectory, I understand that all the harm that I caused was not intentional, but just a means of survival. I hope that this can be fixed some day. “I’m not able to breathe anymore…”

“In blaming kissing bugs for the spread of this horrible disease, we have to analyze humans’ role in history in an impartial manner. In trying to survive, we often adopt thoughtless attitudes that don’t hurt just us.”
